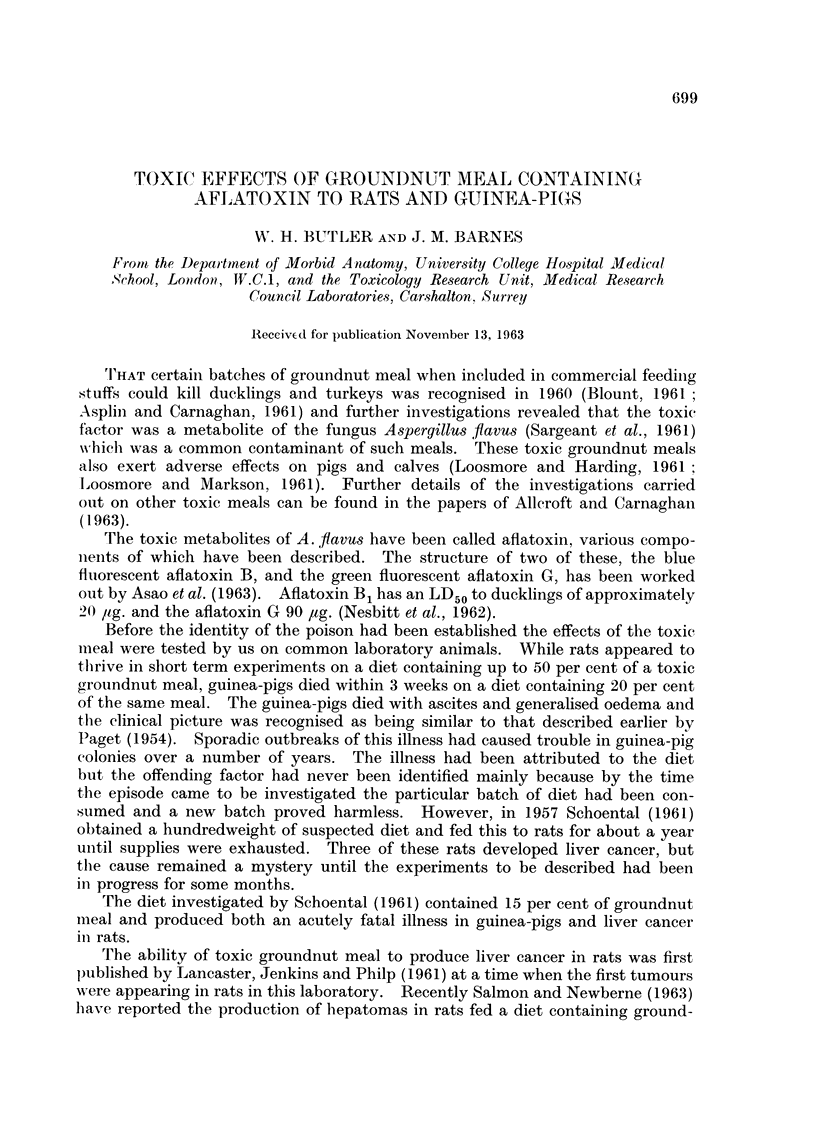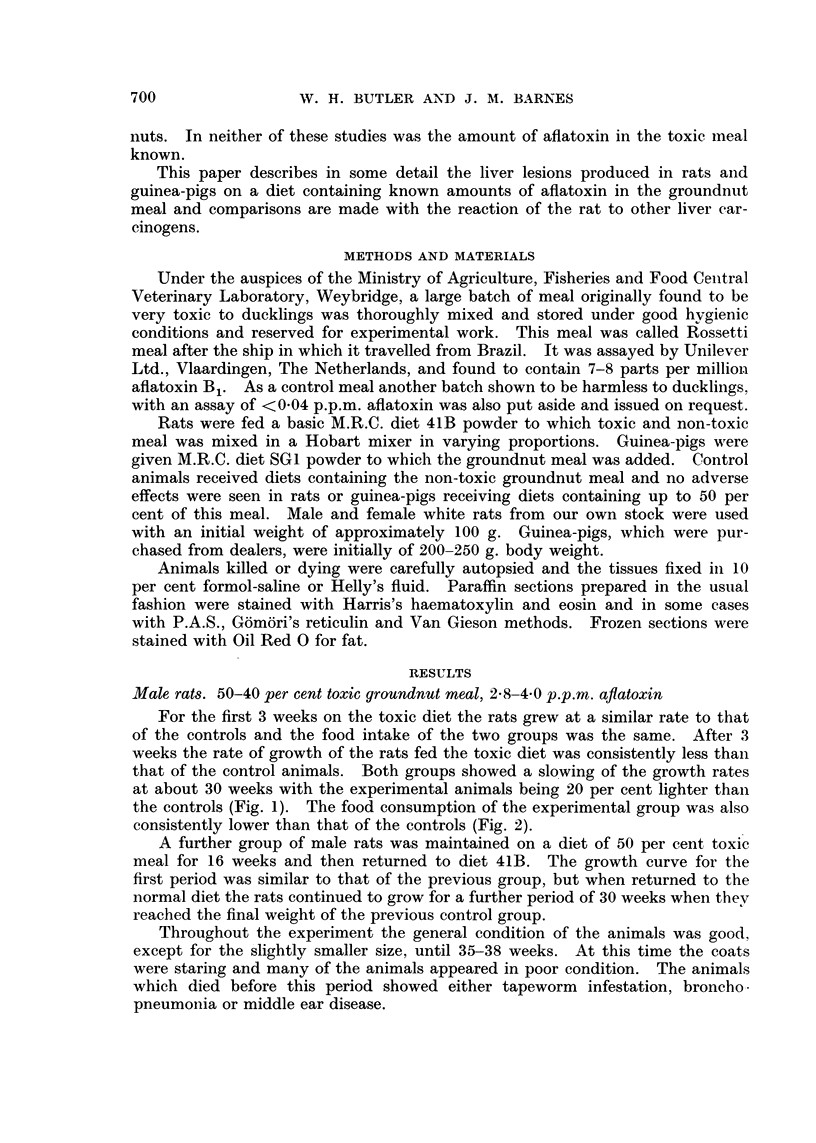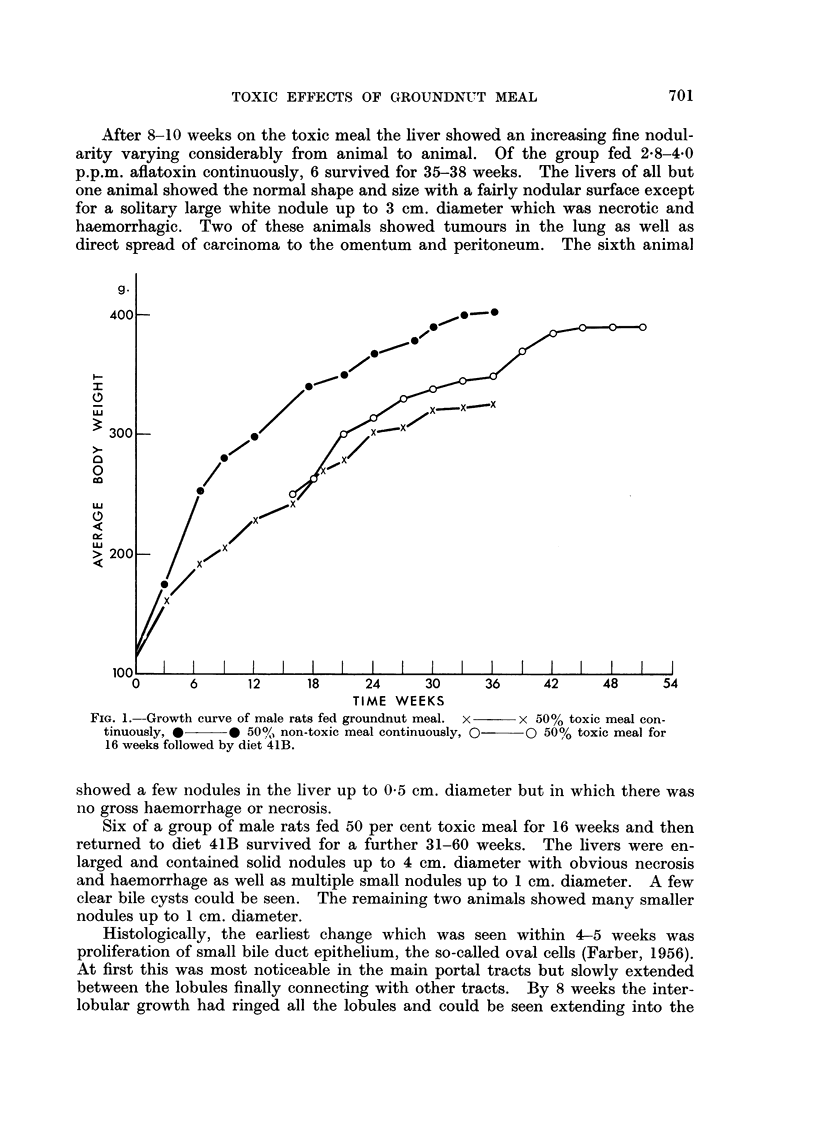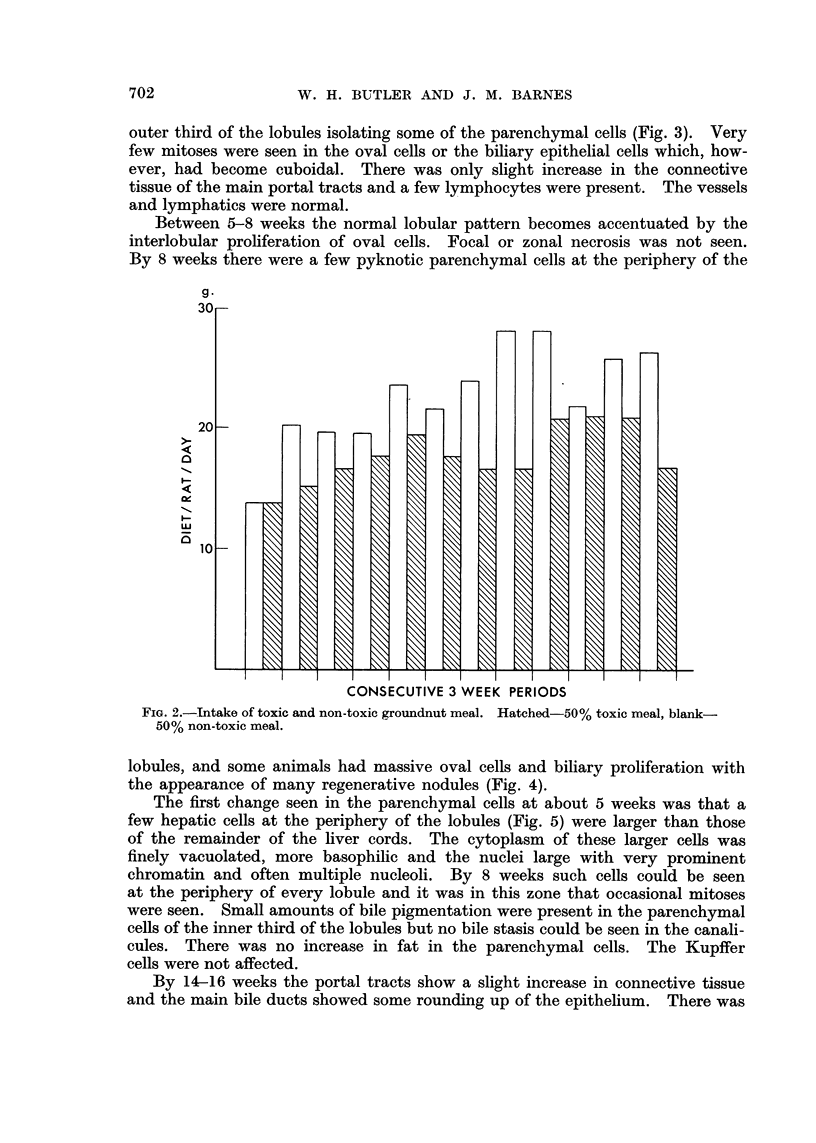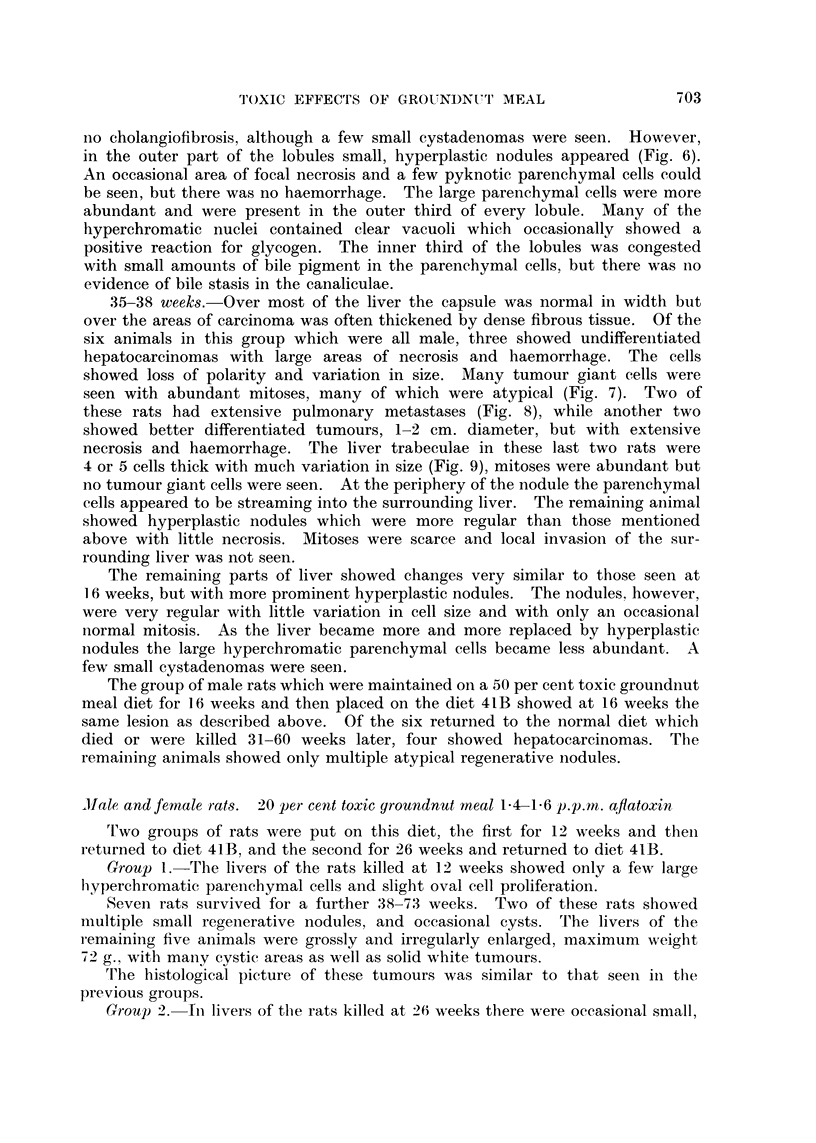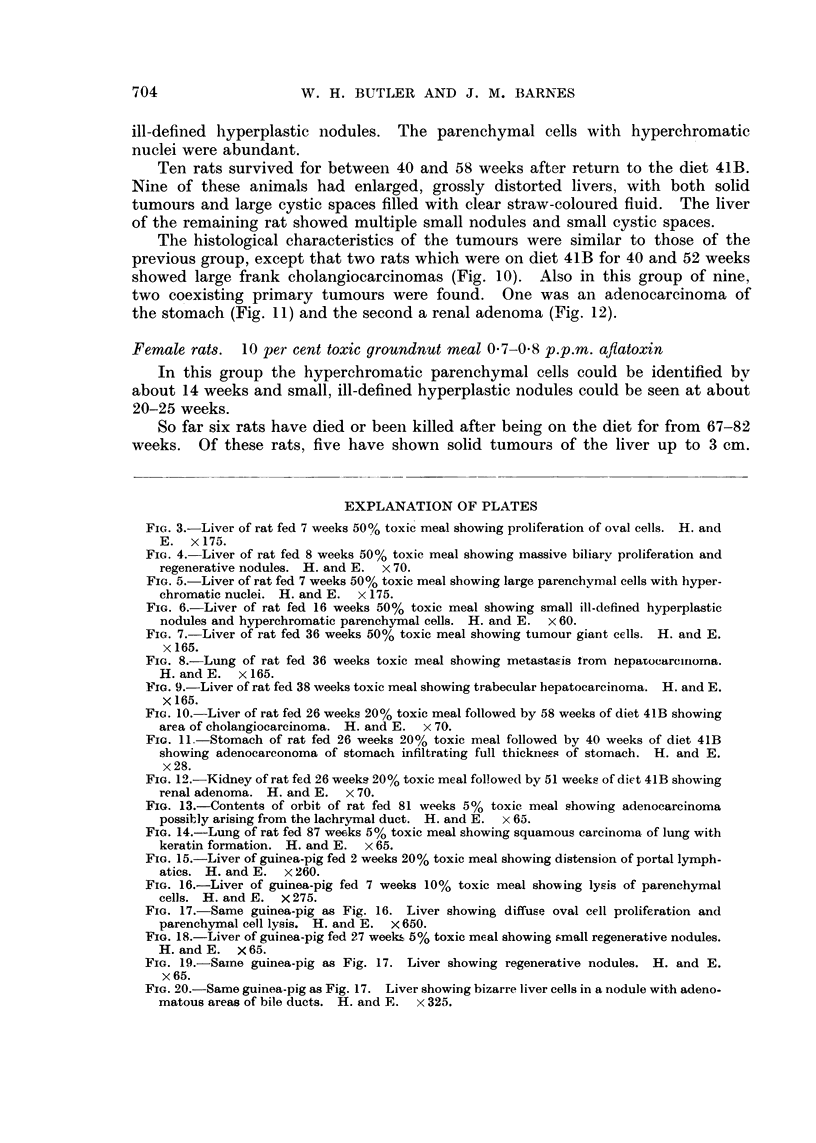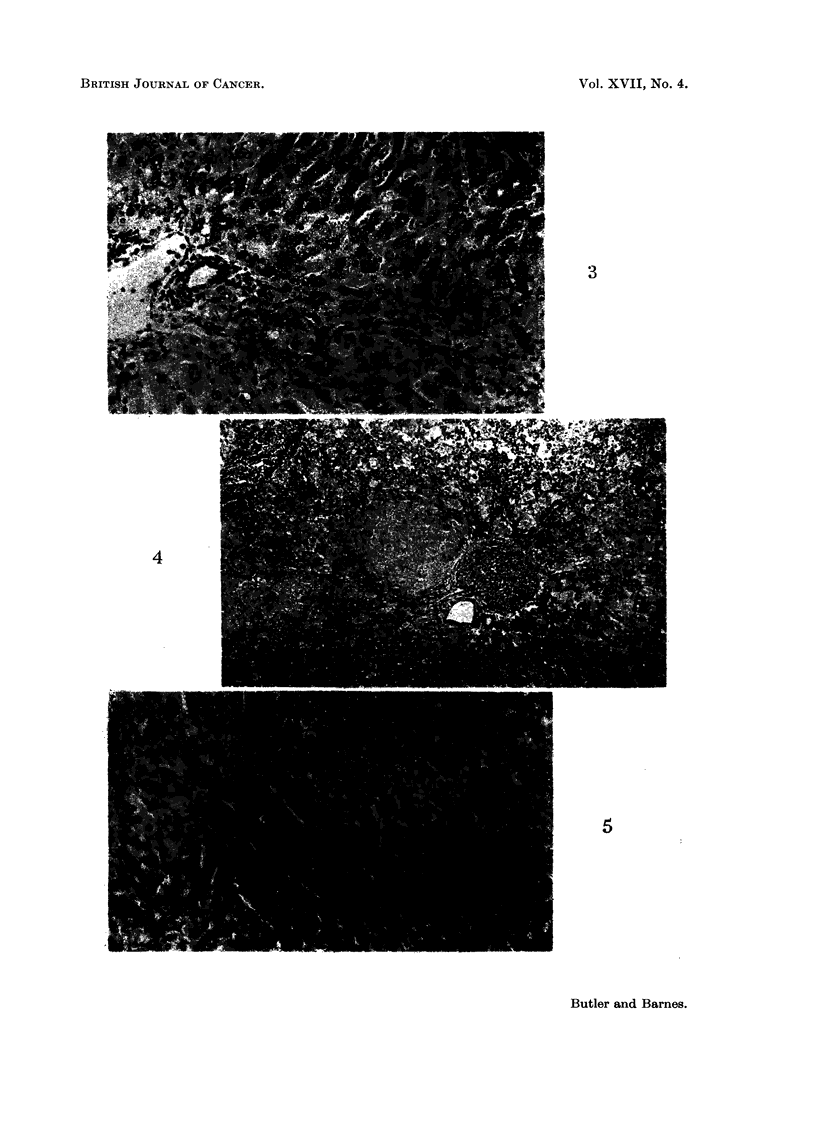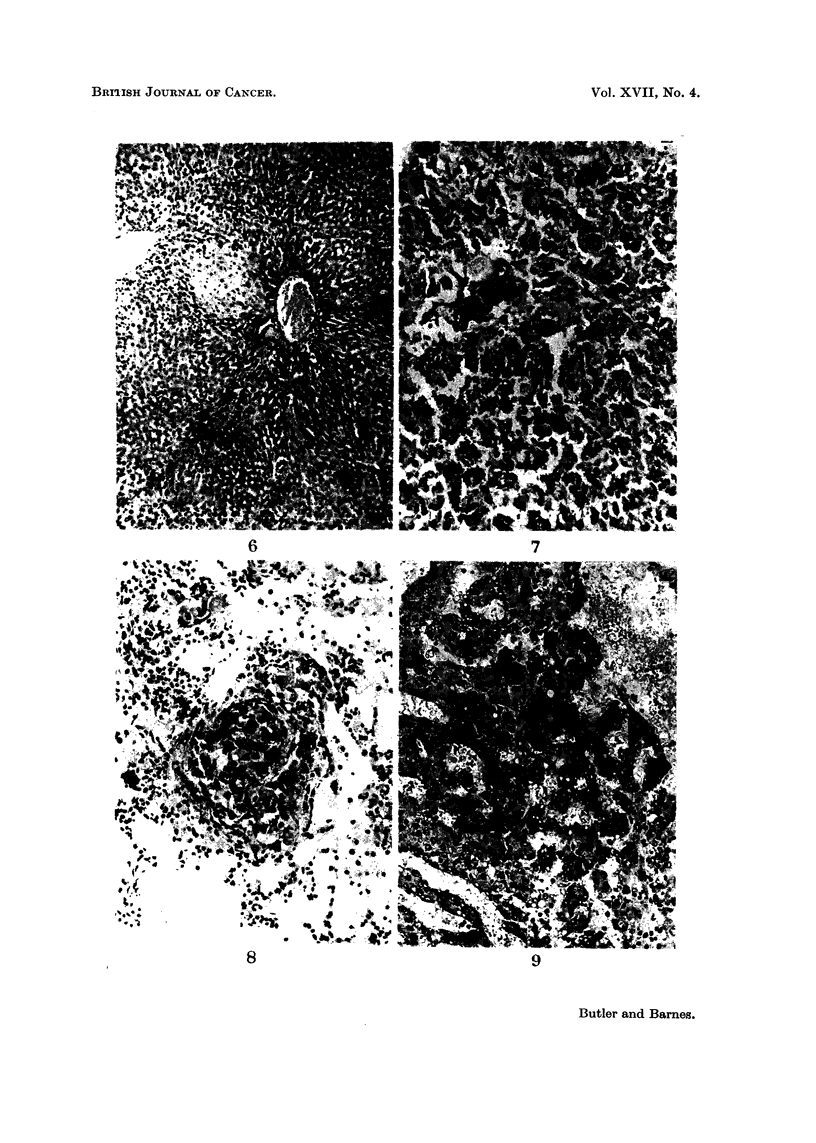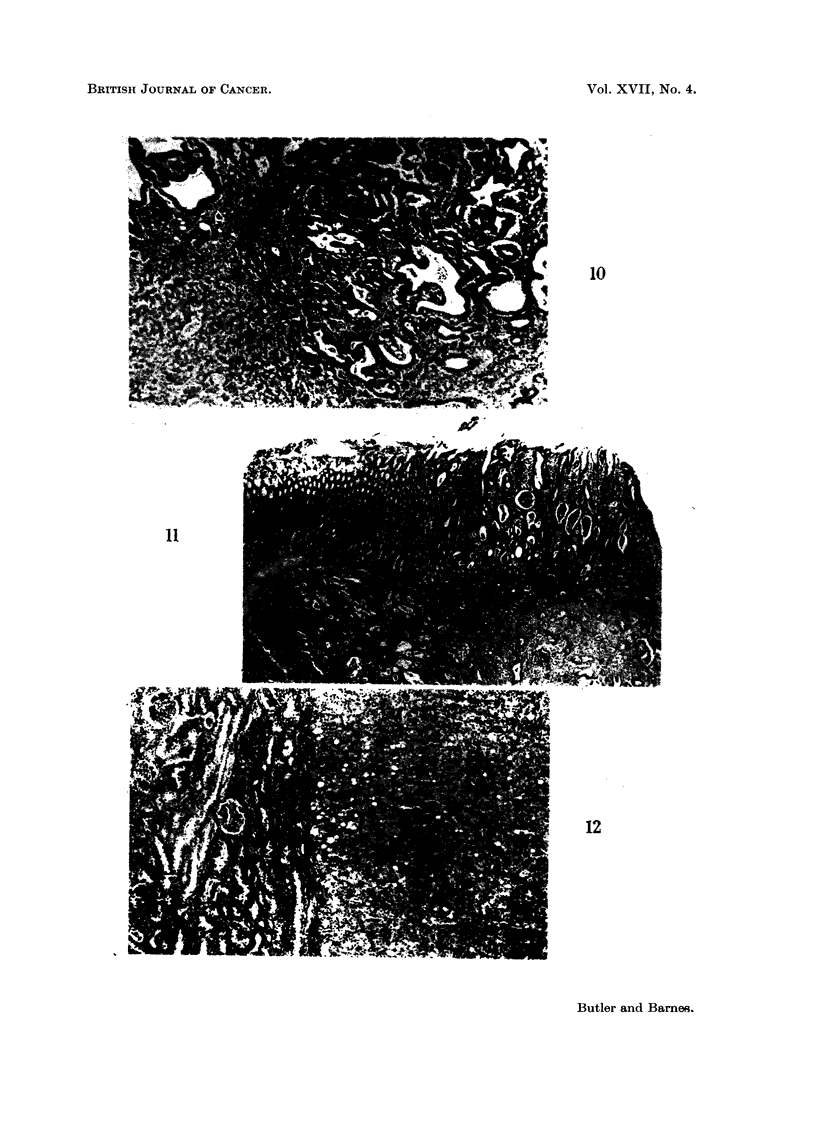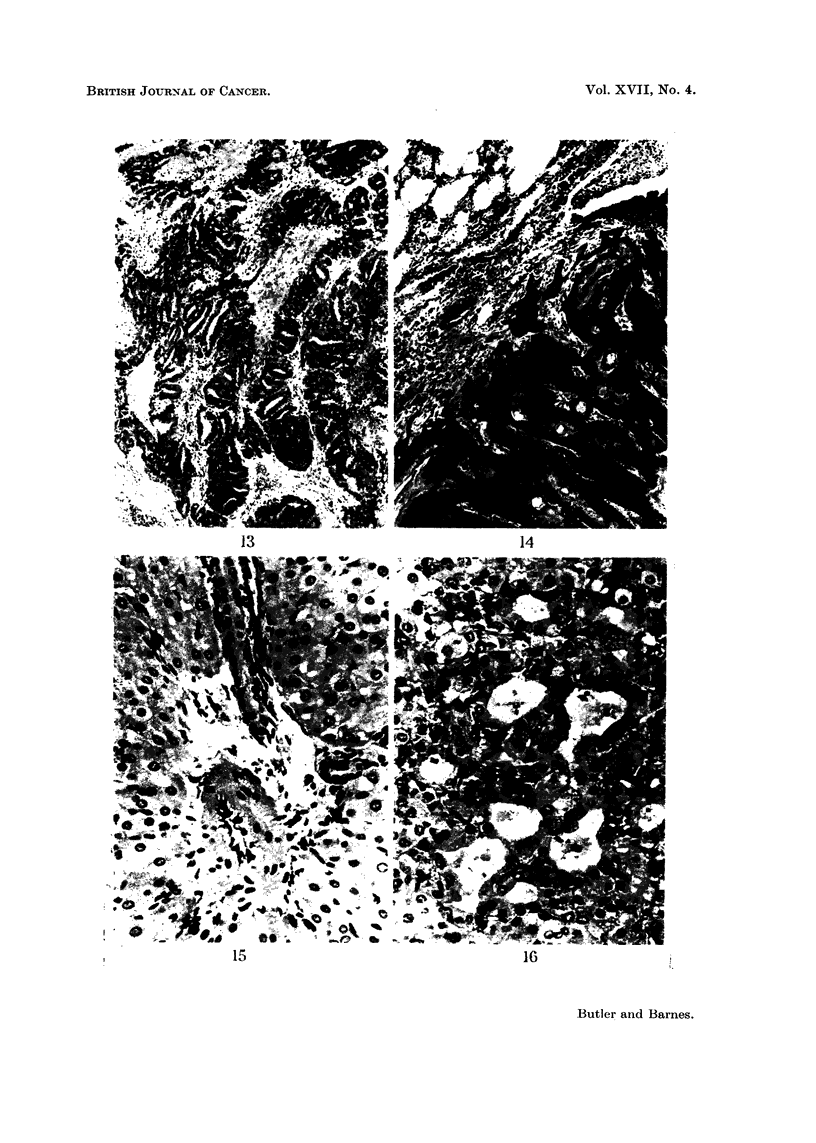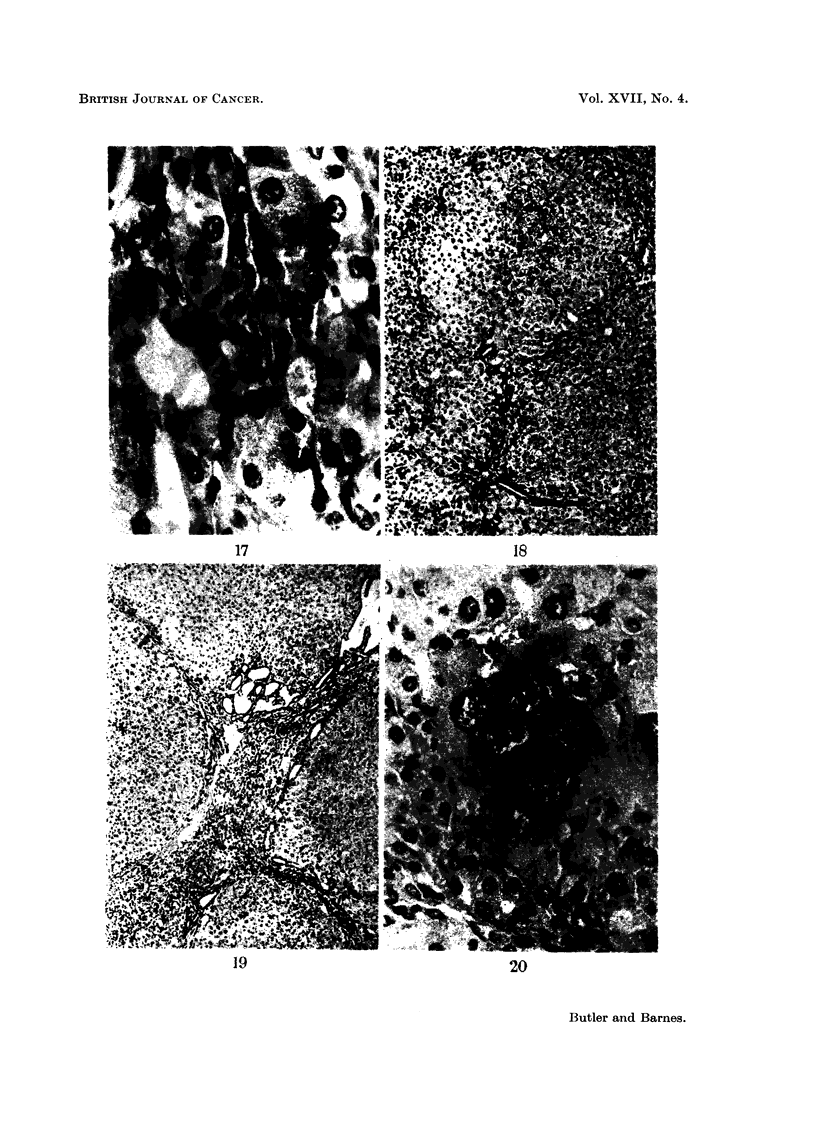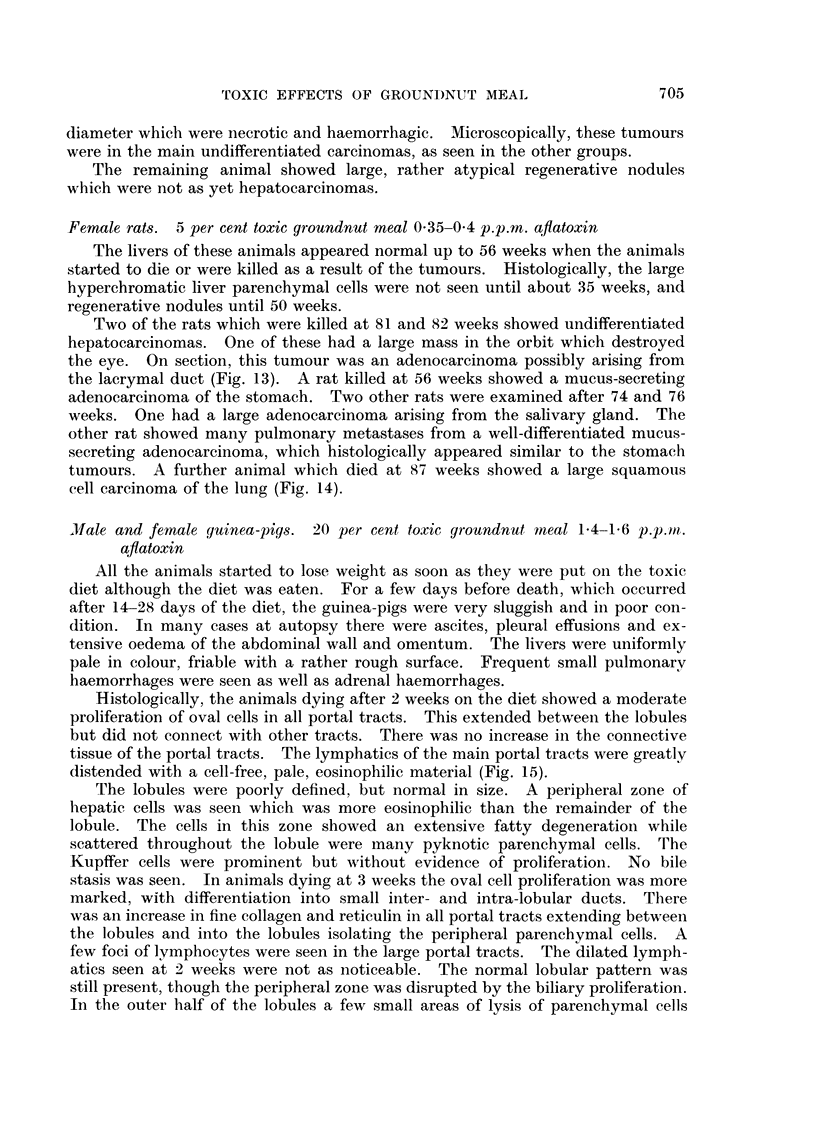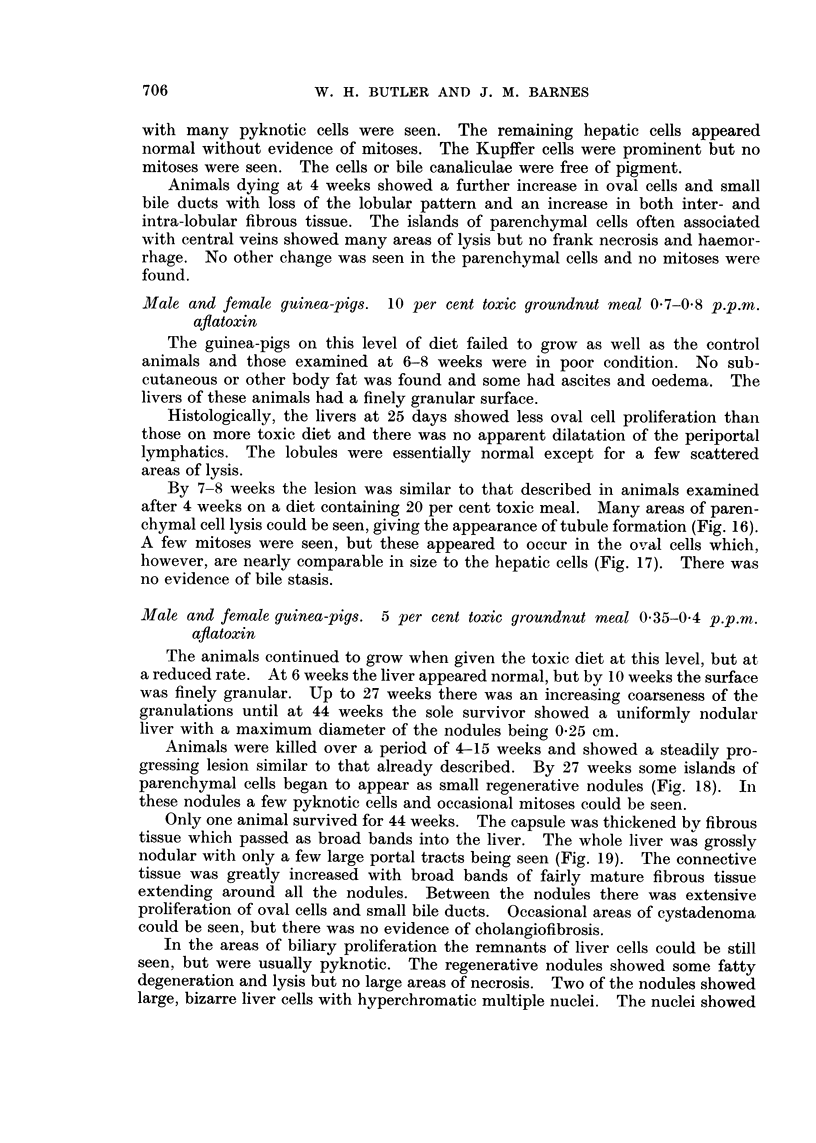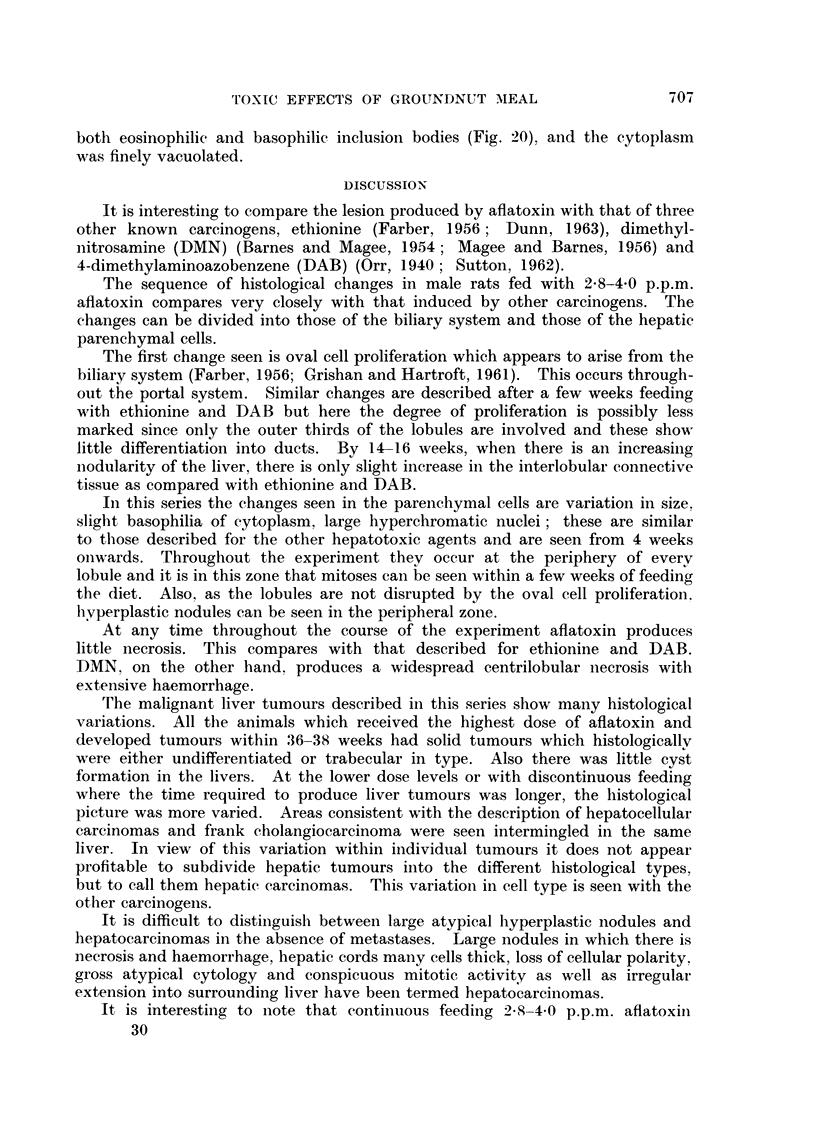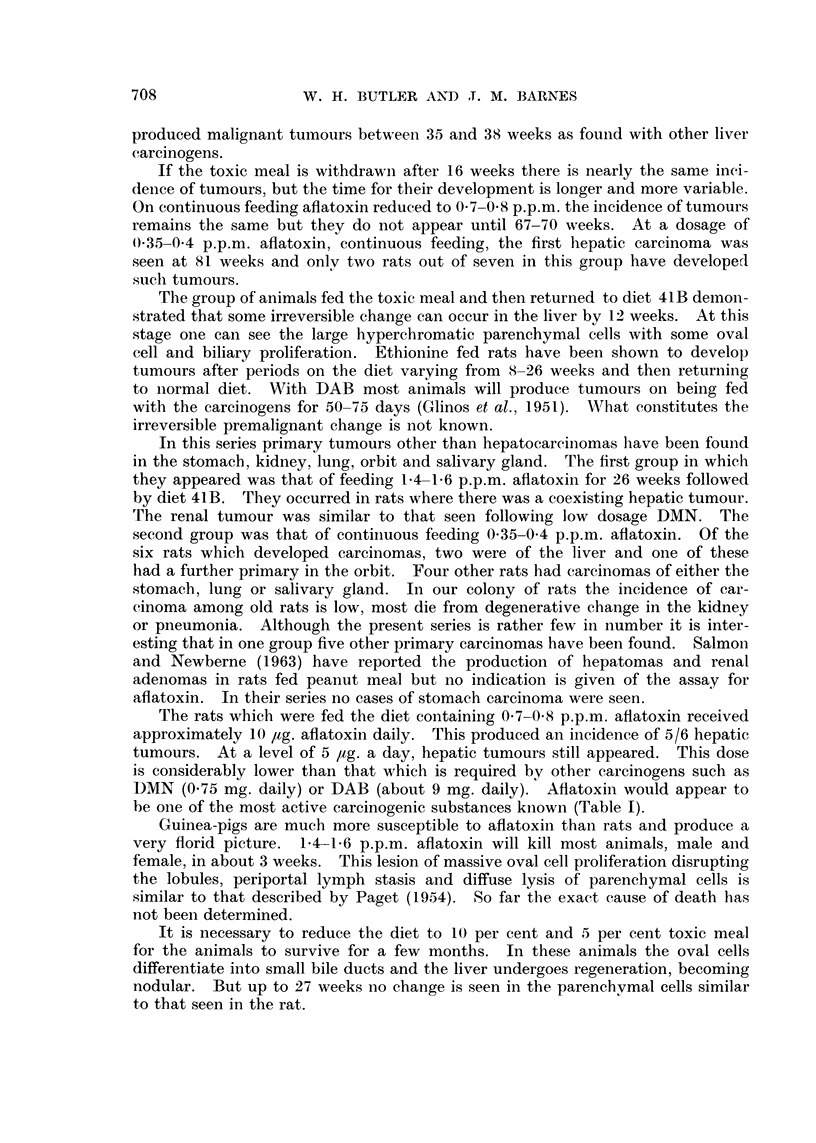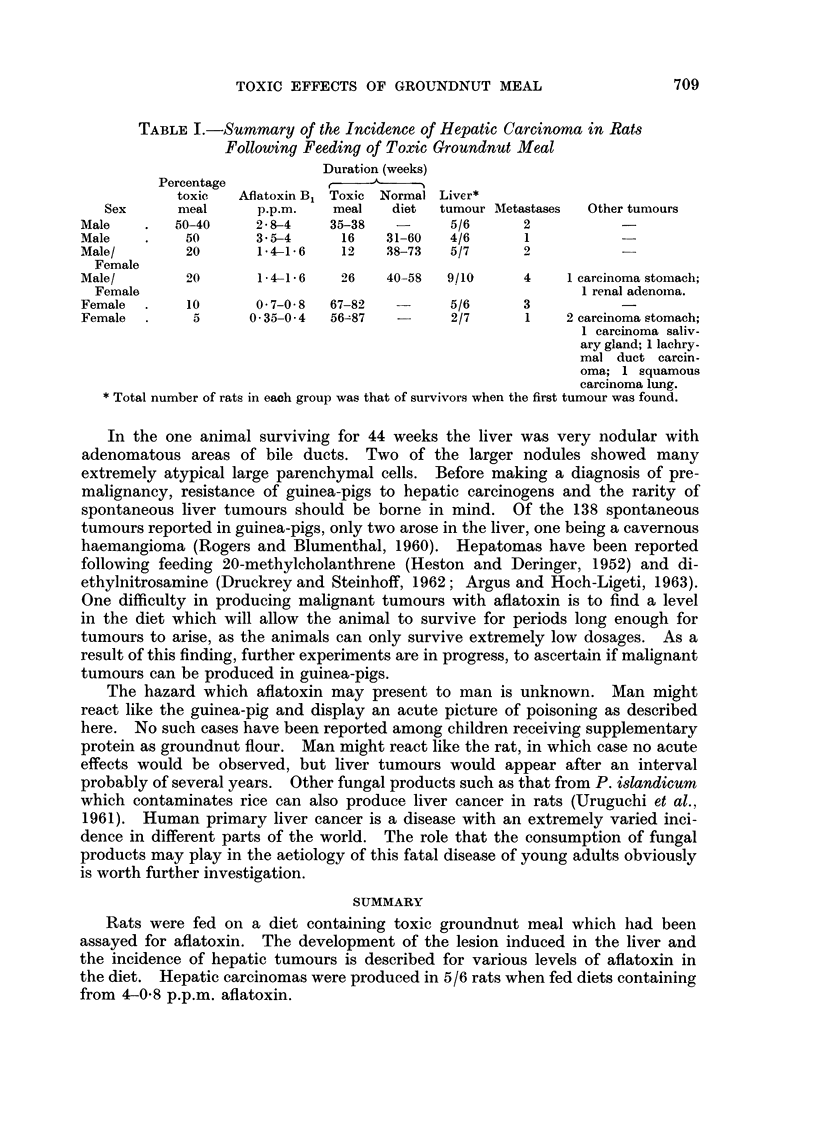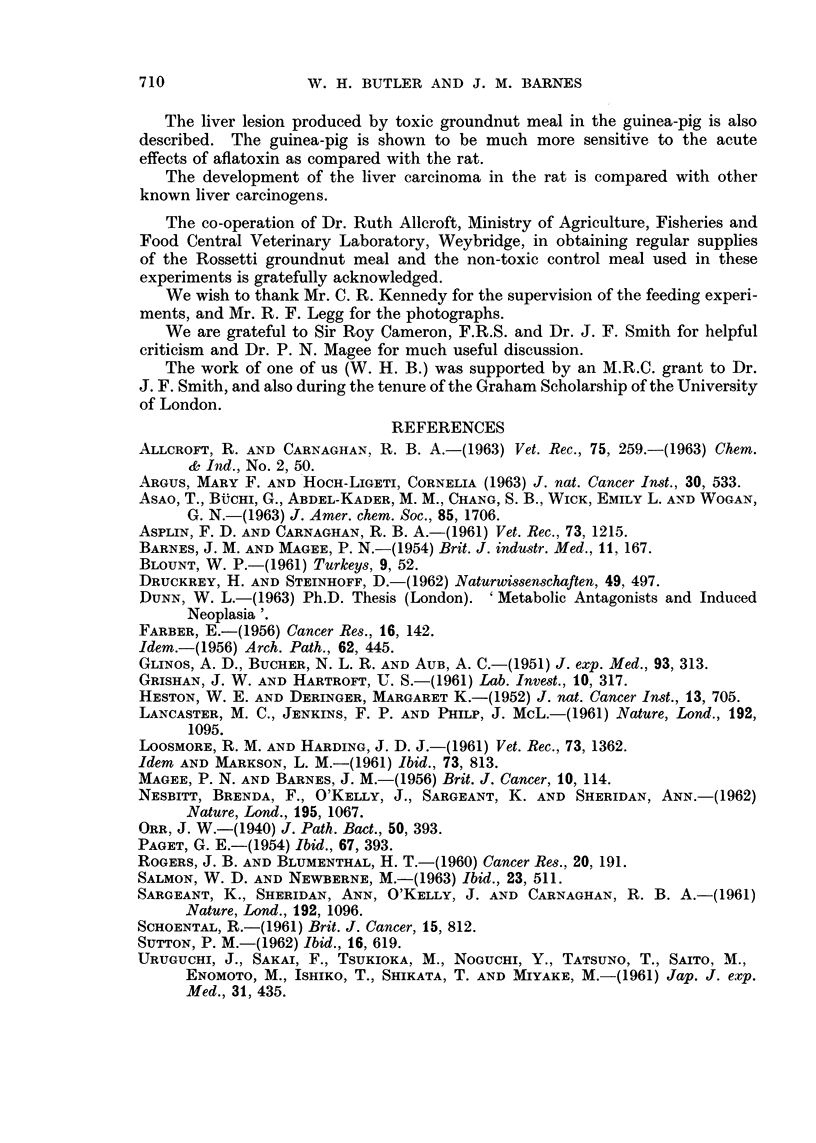# Toxic Effects of Groundnut Meal Containing Aflatoxin to Rats and Guinea-Pigs

**DOI:** 10.1038/bjc.1963.89

**Published:** 1963-12

**Authors:** W. H. Butler, J. M. Barnes

## Abstract

**Images:**


### TOXIC EFFECTS OF GROUNDNUT MEAL CONTAINING AFLATOXIN TO RATS AND GUINEA-PIGS

W. H. BUTLER AND J. M. BARNES

*From the Department of Morbid Anatomy, University College Hospital Medical School, London, W.C.1, and the Toxicology Research Unit, Medical Research Council Laboratories, Carshalton, Surrey*

Received for publication November 13, 1963

THAT certain batches of groundnut meal when included in commercial feeding stuffs could kill ducklings and turkeys was recognised in 1960 (Blount, 1961; Asplin and Carnaghan, 1961) and further investigations revealed that the toxic factor was a metabolite of the fungus *Aspergillus flavus* (Sargeant *et al.*, 1961) which was a common contaminant of such meals. These toxic groundnut meals also exert adverse effects on pigs and calves (Loosmore and Harding, 1961; Loosmore and Markson, 1961). Further details of the investigations carried out on other toxic meals can be found in the papers of Allcroft and Carnaghan (1963).

The toxic metabolites of *A. flavus* have been called aflatoxin, various components of which have been described. The structure of two of these, the blue fluorescent aflatoxin B, and the green fluorescent aflatoxin G, has been worked out by Asao *et al.* (1963). Aflatoxin $B_1$ has an $LD_{50}$ to ducklings of approximately 20 $\mu$g. and the aflatoxin G 90 $\mu$g. (Nesbitt *et al.*, 1962).

Before the identity of the poison had been established the effects of the toxic meal were tested by us on common laboratory animals. While rats appeared to thrive in short term experiments on a diet containing up to 50 per cent of a toxic groundnut meal, guinea-pigs died within 3 weeks on a diet containing 20 per cent of the same meal. The guinea-pigs died with ascites and generalised oedema and the clinical picture was recognised as being similar to that described earlier by Paget (1954). Sporadic outbreaks of this illness had caused trouble in guinea-pig colonies over a number of years. The illness had been attributed to the diet but the offending factor had never been identified mainly because by the time the episode came to be investigated the particular batch of diet had been consumed and a new batch proved harmless. However, in 1957 Schoental (1961) obtained a hundredweight of suspected diet and fed this to rats for about a year until supplies were exhausted. Three of these rats developed liver cancer, but the cause remained a mystery until the experiments to be described had been in progress for some months.

The diet investigated by Schoental (1961) contained 15 per cent of groundnut meal and produced both an acutely fatal illness in guinea-pigs and liver cancer in rats.

The ability of toxic groundnut meal to produce liver cancer in rats was first published by Lancaster, Jenkins and Philp (1961) at a time when the first tumours were appearing in rats in this laboratory. Recently Salmon and Newberne (1963) have reported the production of hepatomas in rats fed a diet containing groundnuts. In neither of these studies was the amount of aflatoxin in the toxic meal known.

This paper describes in some detail the liver lesions produced in rats and guinea-pigs on a diet containing known amounts of aflatoxin in the groundnut meal and comparisons are made with the reaction of the rat to other liver carcinogens.

### METHODS AND MATERIALS

Under the auspices of the Ministry of Agriculture, Fisheries and Food Central Veterinary Laboratory, Weybridge, a large batch of meal originally found to be very toxic to ducklings was thoroughly mixed and stored under good hygienic conditions and reserved for experimental work. This meal was called Rossetti meal after the ship in which it travelled from Brazil. It was assayed by Unilever Ltd., Vlaardingen, The Netherlands, and found to contain 7–8 parts per million aflatoxin $B_1$. As a control meal another batch shown to be harmless to ducklings, with an assay of $<0{\cdot}04$ p.p.m. aflatoxin was also put aside and issued on request.

Rats were fed a basic M.R.C. diet 41B powder to which toxic and non-toxic meal was mixed in a Hobart mixer in varying proportions. Guinea-pigs were given M.R.C. diet SG1 powder to which the groundnut meal was added. Control animals received diets containing the non-toxic groundnut meal and no adverse effects were seen in rats or guinea-pigs receiving diets containing up to 50 per cent of this meal. Male and female white rats from our own stock were used with an initial weight of approximately 100 g. Guinea-pigs, which were purchased from dealers, were initially of 200–250 g. body weight.

Animals killed or dying were carefully autopsied and the tissues fixed in 10 per cent formol-saline or Helly's fluid. Paraffin sections prepared in the usual fashion were stained with Harris's haematoxylin and eosin and in some cases with P.A.S., Gömöri's reticulin and Van Gieson methods. Frozen sections were stained with Oil Red O for fat.

### RESULTS

### *Male rats.* 50–40 *per cent toxic groundnut meal,* 2·8–4·0 *p.p.m. aflatoxin*

For the first 3 weeks on the toxic diet the rats grew at a similar rate to that of the controls and the food intake of the two groups was the same. After 3 weeks the rate of growth of the rats fed the toxic diet was consistently less than that of the control animals. Both groups showed a slowing of the growth rates at about 30 weeks with the experimental animals being 20 per cent lighter than the controls (Fig. 1). The food consumption of the experimental group was also consistently lower than that of the controls (Fig. 2).

A further group of male rats was maintained on a diet of 50 per cent toxic meal for 16 weeks and then returned to diet 41B. The growth curve for the first period was similar to that of the previous group, but when returned to the normal diet the rats continued to grow for a further period of 30 weeks when they reached the final weight of the previous control group.

Throughout the experiment the general condition of the animals was good, except for the slightly smaller size, until 35–38 weeks. At this time the coats were staring and many of the animals appeared in poor condition. The animals which died before this period showed either tapeworm infestation, broncho-pneumonia or middle ear disease.

After 8–10 weeks on the toxic meal the liver showed an increasing fine nodularity varying considerably from animal to animal. Of the group fed 2·8–4·0 p.p.m. aflatoxin continuously, 6 survived for 35–38 weeks. The livers of all but one animal showed the normal shape and size with a fairly nodular surface except for a solitary large white nodule up to 3 cm. diameter which was necrotic and haemorrhagic. Two of these animals showed tumours in the lung as well as direct spread of carcinoma to the omentum and peritoneum. The sixth animal

Fig. 1.—Growth curve of male rats fed groundnut meal. ×——× 50% toxic meal continuously, ●——● 50% non-toxic meal continuously, ○——○ 50% toxic meal for 16 weeks followed by diet 41B.

showed a few nodules in the liver up to 0·5 cm. diameter but in which there was no gross haemorrhage or necrosis.

Six of a group of male rats fed 50 per cent toxic meal for 16 weeks and then returned to diet 41B survived for a further 31–60 weeks. The livers were enlarged and contained solid nodules up to 4 cm. diameter with obvious necrosis and haemorrhage as well as multiple small nodules up to 1 cm. diameter. A few clear bile cysts could be seen. The remaining two animals showed many smaller nodules up to 1 cm. diameter.

Histologically, the earliest change which was seen within 4–5 weeks was proliferation of small bile duct epithelium, the so-called oval cells (Farber, 1956). At first this was most noticeable in the main portal tracts but slowly extended between the lobules finally connecting with other tracts. By 8 weeks the interlobular growth had ringed all the lobules and could be seen extending into the outer third of the lobules isolating some of the parenchymal cells (Fig. 3). Very few mitoses were seen in the oval cells or the biliary epithelial cells which, however, had become cuboidal. There was only slight increase in the connective tissue of the main portal tracts and a few lymphocytes were present. The vessels and lymphatics were normal.

Between 5–8 weeks the normal lobular pattern becomes accentuated by the interlobular proliferation of oval cells. Focal or zonal necrosis was not seen. By 8 weeks there were a few pyknotic parenchymal cells at the periphery of the

Fig. 2.—Intake of toxic and non-toxic groundnut meal. Hatched—50% toxic meal, blank—50% non-toxic meal.

lobules, and some animals had massive oval cells and biliary proliferation with the appearance of many regenerative nodules (Fig. 4).

The first change seen in the parenchymal cells at about 5 weeks was that a few hepatic cells at the periphery of the lobules (Fig. 5) were larger than those of the remainder of the liver cords. The cytoplasm of these larger cells was finely vacuolated, more basophilic and the nuclei large with very prominent chromatin and often multiple nucleoli. By 8 weeks such cells could be seen at the periphery of every lobule and it was in this zone that occasional mitoses were seen. Small amounts of bile pigmentation were present in the parenchymal cells of the inner third of the lobules but no bile stasis could be seen in the canalicules. There was no increase in fat in the parenchymal cells. The Kupffer cells were not affected.

By 14–16 weeks the portal tracts show a slight increase in connective tissue and the main bile ducts showed some rounding up of the epithelium. There was no cholangiofibrosis, although a few small cystadenomas were seen. However, in the outer part of the lobules small, hyperplastic nodules appeared (Fig. 6). An occasional area of focal necrosis and a few pyknotic parenchymal cells could be seen, but there was no haemorrhage. The large parenchymal cells were more abundant and were present in the outer third of every lobule. Many of the hyperchromatic nuclei contained clear vacuoli which occasionally showed a positive reaction for glycogen. The inner third of the lobules was congested with small amounts of bile pigment in the parenchymal cells, but there was no evidence of bile stasis in the canaliculae.

35–38 *weeks*.—Over most of the liver the capsule was normal in width but over the areas of carcinoma was often thickened by dense fibrous tissue. Of the six animals in this group which were all male, three showed undifferentiated hepatocarcinomas with large areas of necrosis and haemorrhage. The cells showed loss of polarity and variation in size. Many tumour giant cells were seen with abundant mitoses, many of which were atypical (Fig. 7). Two of these rats had extensive pulmonary metastases (Fig. 8), while another two showed better differentiated tumours, 1–2 cm. diameter, but with extensive necrosis and haemorrhage. The liver trabeculae in these last two rats were 4 or 5 cells thick with much variation in size (Fig. 9), mitoses were abundant but no tumour giant cells were seen. At the periphery of the nodule the parenchymal cells appeared to be streaming into the surrounding liver. The remaining animal showed hyperplastic nodules which were more regular than those mentioned above with little necrosis. Mitoses were scarce and local invasion of the surrounding liver was not seen.

The remaining parts of liver showed changes very similar to those seen at 16 weeks, but with more prominent hyperplastic nodules. The nodules, however, were very regular with little variation in cell size and with only an occasional normal mitosis. As the liver became more and more replaced by hyperplastic nodules the large hyperchromatic parenchymal cells became less abundant. A few small cystadenomas were seen.

The group of male rats which were maintained on a 50 per cent toxic groundnut meal diet for 16 weeks and then placed on the diet 41B showed at 16 weeks the same lesion as described above. Of the six returned to the normal diet which died or were killed 31–60 weeks later, four showed hepatocarcinomas. The remaining animals showed only multiple atypical regenerative nodules.

*Male and female rats. 20 per cent toxic groundnut meal* 1·4–1·6 *p.p.m. aflatoxin*

Two groups of rats were put on this diet, the first for 12 weeks and then returned to diet 41B, and the second for 26 weeks and returned to diet 41B.

*Group* 1.—The livers of the rats killed at 12 weeks showed only a few large hyperchromatic parenchymal cells and slight oval cell proliferation.

Seven rats survived for a further 38–73 weeks. Two of these rats showed multiple small regenerative nodules, and occasional cysts. The livers of the remaining five animals were grossly and irregularly enlarged, maximum weight 72 g., with many cystic areas as well as solid white tumours.

The histological picture of these tumours was similar to that seen in the previous groups.

*Group* 2.—In livers of the rats killed at 26 weeks there were occasional small, ill-defined hyperplastic nodules. The parenchymal cells with hyperchromatic nuclei were abundant.

Ten rats survived for between 40 and 58 weeks after return to the diet 41B. Nine of these animals had enlarged, grossly distorted livers, with both solid tumours and large cystic spaces filled with clear straw-coloured fluid. The liver of the remaining rat showed multiple small nodules and small cystic spaces.

The histological characteristics of the tumours were similar to those of the previous group, except that two rats which were on diet 41B for 40 and 52 weeks showed large frank cholangiocarcinomas (Fig. 10). Also in this group of nine, two coexisting primary tumours were found. One was an adenocarcinoma of the stomach (Fig. 11) and the second a renal adenoma (Fig. 12).

*Female rats.* 10 *per cent toxic groundnut meal* 0·7–0·8 *p.p.m. aflatoxin*

In this group the hyperchromatic parenchymal cells could be identified by about 14 weeks and small, ill-defined hyperplastic nodules could be seen at about 20–25 weeks.

So far six rats have died or been killed after being on the diet for from 67–82 weeks. Of these rats, five have shown solid tumours of the liver up to 3 cm.

---

EXPLANATION OF PLATES

FIG. 3.—Liver of rat fed 7 weeks 50% toxic meal showing proliferation of oval cells. H. and E. ×175.

FIG. 4.—Liver of rat fed 8 weeks 50% toxic meal showing massive biliary proliferation and regenerative nodules. H. and E. ×70.

FIG. 5.—Liver of rat fed 7 weeks 50% toxic meal showing large parenchymal cells with hyperchromatic nuclei. H. and E. ×175.

FIG. 6.—Liver of rat fed 16 weeks 50% toxic meal showing small ill-defined hyperplastic nodules and hyperchromatic parenchymal cells. H. and E. ×60.

FIG. 7.—Liver of rat fed 36 weeks 50% toxic meal showing tumour giant cells. H. and E. ×165.

FIG. 8.—Lung of rat fed 36 weeks toxic meal showing metastasis from hepatocarcinoma. H. and E. ×165.

FIG. 9.—Liver of rat fed 38 weeks toxic meal showing trabecular hepatocarcinoma. H. and E. ×165.

FIG. 10.—Liver of rat fed 26 weeks 20% toxic meal followed by 58 weeks of diet 41B showing area of cholangiocarcinoma. H. and E. ×70.

FIG. 11.—Stomach of rat fed 26 weeks 20% toxic meal followed by 40 weeks of diet 41B showing adenocarconoma of stomach infiltrating full thickness of stomach. H. and E. ×28.

FIG. 12.—Kidney of rat fed 26 weeks 20% toxic meal followed by 51 weeks of diet 41B showing renal adenoma. H. and E. ×70.

FIG. 13.—Contents of orbit of rat fed 81 weeks 5% toxic meal showing adenocarcinoma possibly arising from the lachrymal duct. H. and E. ×65.

FIG. 14.—Lung of rat fed 87 weeks 5% toxic meal showing squamous carcinoma of lung with keratin formation. H. and E. ×65.

FIG. 15.—Liver of guinea-pig fed 2 weeks 20% toxic meal showing distension of portal lymphatics. H. and E. ×260.

FIG. 16.—Liver of guinea-pig fed 7 weeks 10% toxic meal showing lysis of parenchymal cells. H. and E. ×275.

FIG. 17.—Same guinea-pig as Fig. 16. Liver showing diffuse oval cell proliferation and parenchymal cell lysis. H. and E. ×650.

FIG. 18.—Liver of guinea-pig fed 27 weeks 5% toxic meal showing small regenerative nodules. H. and E. ×65.

FIG. 19.—Same guinea-pig as Fig. 17. Liver showing regenerative nodules. H. and E. ×65.

FIG. 20.—Same guinea-pig as Fig. 17. Liver showing bizarre liver cells in a nodule with adenomatous areas of bile ducts. H. and E. ×325.

3

4

5

Butler and Barnes.

6

7

8

9

10

11

12

Butler and Barnes.

13

14

15

16

Butler and Barnes.

17

18

19

20

Butler and Barnes.

diameter which were necrotic and haemorrhagic. Microscopically, these tumours were in the main undifferentiated carcinomas, as seen in the other groups.

The remaining animal showed large, rather atypical regenerative nodules which were not as yet hepatocarcinomas.

*Female rats. 5 per cent toxic groundnut meal* 0·35–0·4 *p.p.m. aflatoxin*

The livers of these animals appeared normal up to 56 weeks when the animals started to die or were killed as a result of the tumours. Histologically, the large hyperchromatic liver parenchymal cells were not seen until about 35 weeks, and regenerative nodules until 50 weeks.

Two of the rats which were killed at 81 and 82 weeks showed undifferentiated hepatocarcinomas. One of these had a large mass in the orbit which destroyed the eye. On section, this tumour was an adenocarcinoma possibly arising from the lacrymal duct (Fig. 13). A rat killed at 56 weeks showed a mucus-secreting adenocarcinoma of the stomach. Two other rats were examined after 74 and 76 weeks. One had a large adenocarcinoma arising from the salivary gland. The other rat showed many pulmonary metastases from a well-differentiated mucus-secreting adenocarcinoma, which histologically appeared similar to the stomach tumours. A further animal which died at 87 weeks showed a large squamous cell carcinoma of the lung (Fig. 14).

*Male and female guinea-pigs. 20 per cent toxic groundnut meal* 1·4–1·6 *p.p.m. aflatoxin*

All the animals started to lose weight as soon as they were put on the toxic diet although the diet was eaten. For a few days before death, which occurred after 14–28 days of the diet, the guinea-pigs were very sluggish and in poor condition. In many cases at autopsy there were ascites, pleural effusions and extensive oedema of the abdominal wall and omentum. The livers were uniformly pale in colour, friable with a rather rough surface. Frequent small pulmonary haemorrhages were seen as well as adrenal haemorrhages.

Histologically, the animals dying after 2 weeks on the diet showed a moderate proliferation of oval cells in all portal tracts. This extended between the lobules but did not connect with other tracts. There was no increase in the connective tissue of the portal tracts. The lymphatics of the main portal tracts were greatly distended with a cell-free, pale, eosinophilic material (Fig. 15).

The lobules were poorly defined, but normal in size. A peripheral zone of hepatic cells was seen which was more eosinophilic than the remainder of the lobule. The cells in this zone showed an extensive fatty degeneration while scattered throughout the lobule were many pyknotic parenchymal cells. The Kupffer cells were prominent but without evidence of proliferation. No bile stasis was seen. In animals dying at 3 weeks the oval cell proliferation was more marked, with differentiation into small inter- and intra-lobular ducts. There was an increase in fine collagen and reticulin in all portal tracts extending between the lobules and into the lobules isolating the peripheral parenchymal cells. A few foci of lymphocytes were seen in the large portal tracts. The dilated lymphatics seen at 2 weeks were not as noticeable. The normal lobular pattern was still present, though the peripheral zone was disrupted by the biliary proliferation. In the outer half of the lobules a few small areas of lysis of parenchymal cells with many pyknotic cells were seen. The remaining hepatic cells appeared normal without evidence of mitoses. The Kupffer cells were prominent but no mitoses were seen. The cells or bile canaliculae were free of pigment.

Animals dying at 4 weeks showed a further increase in oval cells and small bile ducts with loss of the lobular pattern and an increase in both inter- and intra-lobular fibrous tissue. The islands of parenchymal cells often associated with central veins showed many areas of lysis but no frank necrosis and haemorrhage. No other change was seen in the parenchymal cells and no mitoses were found.

*Male and female guinea-pigs. 10 per cent toxic groundnut meal* 0·7–0·8 *p.p.m. aflatoxin*

The guinea-pigs on this level of diet failed to grow as well as the control animals and those examined at 6–8 weeks were in poor condition. No subcutaneous or other body fat was found and some had ascites and oedema. The livers of these animals had a finely granular surface.

Histologically, the livers at 25 days showed less oval cell proliferation than those on more toxic diet and there was no apparent dilatation of the periportal lymphatics. The lobules were essentially normal except for a few scattered areas of lysis.

By 7–8 weeks the lesion was similar to that described in animals examined after 4 weeks on a diet containing 20 per cent toxic meal. Many areas of parenchymal cell lysis could be seen, giving the appearance of tubule formation (Fig. 16). A few mitoses were seen, but these appeared to occur in the oval cells which, however, are nearly comparable in size to the hepatic cells (Fig. 17). There was no evidence of bile stasis.

*Male and female guinea-pigs. 5 per cent toxic groundnut meal* 0·35–0·4 *p.p.m. aflatoxin*

The animals continued to grow when given the toxic diet at this level, but at a reduced rate. At 6 weeks the liver appeared normal, but by 10 weeks the surface was finely granular. Up to 27 weeks there was an increasing coarseness of the granulations until at 44 weeks the sole survivor showed a uniformly nodular liver with a maximum diameter of the nodules being 0·25 cm.

Animals were killed over a period of 4–15 weeks and showed a steadily progressing lesion similar to that already described. By 27 weeks some islands of parenchymal cells began to appear as small regenerative nodules (Fig. 18). In these nodules a few pyknotic cells and occasional mitoses could be seen.

Only one animal survived for 44 weeks. The capsule was thickened by fibrous tissue which passed as broad bands into the liver. The whole liver was grossly nodular with only a few large portal tracts being seen (Fig. 19). The connective tissue was greatly increased with broad bands of fairly mature fibrous tissue extending around all the nodules. Between the nodules there was extensive proliferation of oval cells and small bile ducts. Occasional areas of cystadenoma could be seen, but there was no evidence of cholangiofibrosis.

In the areas of biliary proliferation the remnants of liver cells could be still seen, but were usually pyknotic. The regenerative nodules showed some fatty degeneration and lysis but no large areas of necrosis. Two of the nodules showed large, bizarre liver cells with hyperchromatic multiple nuclei. The nuclei showed both eosinophilic and basophilic inclusion bodies (Fig. 20), and the cytoplasm was finely vacuolated.

### DISCUSSION

It is interesting to compare the lesion produced by aflatoxin with that of three other known carcinogens, ethionine (Farber, 1956; Dunn, 1963), dimethylnitrosamine (DMN) (Barnes and Magee, 1954; Magee and Barnes, 1956) and 4-dimethylaminoazobenzene (DAB) (Orr, 1940; Sutton, 1962).

The sequence of histological changes in male rats fed with 2·8–4·0 p.p.m. aflatoxin compares very closely with that induced by other carcinogens. The changes can be divided into those of the biliary system and those of the hepatic parenchymal cells.

The first change seen is oval cell proliferation which appears to arise from the biliary system (Farber, 1956; Grishan and Hartroft, 1961). This occurs throughout the portal system. Similar changes are described after a few weeks feeding with ethionine and DAB but here the degree of proliferation is possibly less marked since only the outer thirds of the lobules are involved and these show little differentiation into ducts. By 14–16 weeks, when there is an increasing nodularity of the liver, there is only slight increase in the interlobular connective tissue as compared with ethionine and DAB.

In this series the changes seen in the parenchymal cells are variation in size, slight basophilia of cytoplasm, large hyperchromatic nuclei; these are similar to those described for the other hepatotoxic agents and are seen from 4 weeks onwards. Throughout the experiment they occur at the periphery of every lobule and it is in this zone that mitoses can be seen within a few weeks of feeding the diet. Also, as the lobules are not disrupted by the oval cell proliferation, hyperplastic nodules can be seen in the peripheral zone.

At any time throughout the course of the experiment aflatoxin produces little necrosis. This compares with that described for ethionine and DAB. DMN, on the other hand, produces a widespread centrilobular necrosis with extensive haemorrhage.

The malignant liver tumours described in this series show many histological variations. All the animals which received the highest dose of aflatoxin and developed tumours within 36–38 weeks had solid tumours which histologically were either undifferentiated or trabecular in type. Also there was little cyst formation in the livers. At the lower dose levels or with discontinuous feeding where the time required to produce liver tumours was longer, the histological picture was more varied. Areas consistent with the description of hepatocellular carcinomas and frank cholangiocarcinoma were seen intermingled in the same liver. In view of this variation within individual tumours it does not appear profitable to subdivide hepatic tumours into the different histological types, but to call them hepatic carcinomas. This variation in cell type is seen with the other carcinogens.

It is difficult to distinguish between large atypical hyperplastic nodules and hepatocarcinomas in the absence of metastases. Large nodules in which there is necrosis and haemorrhage, hepatic cords many cells thick, loss of cellular polarity, gross atypical cytology and conspicuous mitotic activity as well as irregular extension into surrounding liver have been termed hepatocarcinomas.

It is interesting to note that continuous feeding 2·8–4·0 p.p.m. aflatoxin produced malignant tumours between 35 and 38 weeks as found with other liver carcinogens.

If the toxic meal is withdrawn after 16 weeks there is nearly the same incidence of tumours, but the time for their development is longer and more variable. On continuous feeding aflatoxin reduced to 0·7–0·8 p.p.m. the incidence of tumours remains the same but they do not appear until 67–70 weeks. At a dosage of 0·35–0·4 p.p.m. aflatoxin, continuous feeding, the first hepatic carcinoma was seen at 81 weeks and only two rats out of seven in this group have developed such tumours.

The group of animals fed the toxic meal and then returned to diet 41B demonstrated that some irreversible change can occur in the liver by 12 weeks. At this stage one can see the large hyperchromatic parenchymal cells with some oval cell and biliary proliferation. Ethionine fed rats have been shown to develop tumours after periods on the diet varying from 8–26 weeks and then returning to normal diet. With DAB most animals will produce tumours on being fed with the carcinogens for 50–75 days (Glinos *et al.*, 1951). What constitutes the irreversible premalignant change is not known.

In this series primary tumours other than hepatocarcinomas have been found in the stomach, kidney, lung, orbit and salivary gland. The first group in which they appeared was that of feeding 1·4–1·6 p.p.m. aflatoxin for 26 weeks followed by diet 41B. They occurred in rats where there was a coexisting hepatic tumour. The renal tumour was similar to that seen following low dosage DMN. The second group was that of continuous feeding 0·35–0·4 p.p.m. aflatoxin. Of the six rats which developed carcinomas, two were of the liver and one of these had a further primary in the orbit. Four other rats had carcinomas of either the stomach, lung or salivary gland. In our colony of rats the incidence of carcinoma among old rats is low, most die from degenerative change in the kidney or pneumonia. Although the present series is rather few in number it is interesting that in one group five other primary carcinomas have been found. Salmon and Newberne (1963) have reported the production of hepatomas and renal adenomas in rats fed peanut meal but no indication is given of the assay for aflatoxin. In their series no cases of stomach carcinoma were seen.

The rats which were fed the diet containing 0·7–0·8 p.p.m. aflatoxin received approximately 10 $\mu$g. aflatoxin daily. This produced an incidence of 5/6 hepatic tumours. At a level of 5 $\mu$g. a day, hepatic tumours still appeared. This dose is considerably lower than that which is required by other carcinogens such as DMN (0·75 mg. daily) or DAB (about 9 mg. daily). Aflatoxin would appear to be one of the most active carcinogenic substances known (Table I).

Guinea-pigs are much more susceptible to aflatoxin than rats and produce a very florid picture. 1·4–1·6 p.p.m. aflatoxin will kill most animals, male and female, in about 3 weeks. This lesion of massive oval cell proliferation disrupting the lobules, periportal lymph stasis and diffuse lysis of parenchymal cells is similar to that described by Paget (1954). So far the exact cause of death has not been determined.

It is necessary to reduce the diet to 10 per cent and 5 per cent toxic meal for the animals to survive for a few months. In these animals the oval cells differentiate into small bile ducts and the liver undergoes regeneration, becoming nodular. But up to 27 weeks no change is seen in the parenchymal cells similar to that seen in the rat.

TABLE I.—*Summary of the Incidence of Hepatic Carcinoma in Rats Following Feeding of Toxic Groundnut Meal*

| Sex | Percentage toxic meal | Aflatoxin $B_1$ p.p.m. | Duration (weeks) Toxic meal | Normal diet | Liver* tumour | Metastases | Other tumours |
|---|---|---|---|---|---|---|---|
| Male | 50–40 | 2·8–4 | 35–38 | — | 5/6 | 2 | — |
| Male | 50 | 3·5–4 | 16 | 31–60 | 4/6 | 1 | — |
| Male/Female | 20 | 1·4–1·6 | 12 | 38–73 | 5/7 | 2 | — |
| Male/Female | 20 | 1·4–1·6 | 26 | 40–58 | 9/10 | 4 | 1 carcinoma stomach; 1 renal adenoma. |
| Female | 10 | 0·7–0·8 | 67–82 | — | 5/6 | 3 | — |
| Female | 5 | 0·35–0·4 | 56–87 | — | 2/7 | 1 | 2 carcinoma stomach; 1 carcinoma salivary gland; 1 lachrymal duct carcinoma; 1 squamous carcinoma lung. |

* Total number of rats in each group was that of survivors when the first tumour was found.

In the one animal surviving for 44 weeks the liver was very nodular with adenomatous areas of bile ducts. Two of the larger nodules showed many extremely atypical large parenchymal cells. Before making a diagnosis of premalignancy, resistance of guinea-pigs to hepatic carcinogens and the rarity of spontaneous liver tumours should be borne in mind. Of the 138 spontaneous tumours reported in guinea-pigs, only two arose in the liver, one being a cavernous haemangioma (Rogers and Blumenthal, 1960). Hepatomas have been reported following feeding 20-methylcholanthrene (Heston and Deringer, 1952) and diethylnitrosamine (Druckrey and Steinhoff, 1962; Argus and Hoch-Ligeti, 1963). One difficulty in producing malignant tumours with aflatoxin is to find a level in the diet which will allow the animal to survive for periods long enough for tumours to arise, as the animals can only survive extremely low dosages. As a result of this finding, further experiments are in progress, to ascertain if malignant tumours can be produced in guinea-pigs.

The hazard which aflatoxin may present to man is unknown. Man might react like the guinea-pig and display an acute picture of poisoning as described here. No such cases have been reported among children receiving supplementary protein as groundnut flour. Man might react like the rat, in which case no acute effects would be observed, but liver tumours would appear after an interval probably of several years. Other fungal products such as that from *P. islandicum* which contaminates rice can also produce liver cancer in rats (Uruguchi *et al.*, 1961). Human primary liver cancer is a disease with an extremely varied incidence in different parts of the world. The role that the consumption of fungal products may play in the aetiology of this fatal disease of young adults obviously is worth further investigation.

### SUMMARY

Rats were fed on a diet containing toxic groundnut meal which had been assayed for aflatoxin. The development of the lesion induced in the liver and the incidence of hepatic tumours is described for various levels of aflatoxin in the diet. Hepatic carcinomas were produced in 5/6 rats when fed diets containing from 4–0·8 p.p.m. aflatoxin.

The liver lesion produced by toxic groundnut meal in the guinea-pig is also described. The guinea-pig is shown to be much more sensitive to the acute effects of aflatoxin as compared with the rat.

The development of the liver carcinoma in the rat is compared with other known liver carcinogens.

The co-operation of Dr. Ruth Allcroft, Ministry of Agriculture, Fisheries and Food Central Veterinary Laboratory, Weybridge, in obtaining regular supplies of the Rossetti groundnut meal and the non-toxic control meal used in these experiments is gratefully acknowledged.

We wish to thank Mr. C. R. Kennedy for the supervision of the feeding experiments, and Mr. R. F. Legg for the photographs.

We are grateful to Sir Roy Cameron, F.R.S. and Dr. J. F. Smith for helpful criticism and Dr. P. N. Magee for much useful discussion.

The work of one of us (W. H. B.) was supported by an M.R.C. grant to Dr. J. F. Smith, and also during the tenure of the Graham Scholarship of the University of London.

### REFERENCES

Allcroft, R. and Carnaghan, R. B. A.—(1963) *Vet. Rec.*, **75**, 259.—(1963) *Chem. & Ind.*, No. 2, 50.
Argus, Mary F. and Hoch-Ligeti, Cornelia (1963) *J. nat. Cancer Inst.*, **30**, 533.
Asao, T., Büchi, G., Abdel-Kader, M. M., Chang, S. B., Wick, Emily L. and Wogan, G. N.—(1963) *J. Amer. chem. Soc.*, **85**, 1706.
Asplin, F. D. and Carnaghan, R. B. A.—(1961) *Vet. Rec.*, **73**, 1215.
Barnes, J. M. and Magee, P. N.—(1954) *Brit. J. industr. Med.*, **11**, 167.
Blount, W. P.—(1961) *Turkeys*, **9**, 52.
Druckrey, H. and Steinhoff, D.—(1962) *Naturwissenschaften*, **49**, 497.
Dunn, W. L.—(1963) Ph.D. Thesis (London). ‘Metabolic Antagonists and Induced Neoplasia’.
Farber, E.—(1956) *Cancer Res.*, **16**, 142.
*Idem.*—(1956) *Arch. Path.*, **62**, 445.
Glinos, A. D., Bucher, N. L. R. and Aub, A. C.—(1951) *J. exp. Med.*, **93**, 313.
Grishan, J. W. and Hartroft, U. S.—(1961) *Lab. Invest.*, **10**, 317.
Heston, W. E. and Deringer, Margaret K.—(1952) *J. nat. Cancer Inst.*, **13**, 705.
Lancaster, M. C., Jenkins, F. P. and Philp, J. McL.—(1961) *Nature, Lond.*, **192**, 1095.
Loosmore, R. M. and Harding, J. D. J.—(1961) *Vet. Rec.*, **73**, 1362.
*Idem* and Markson, L. M.—(1961) *Ibid.*, **73**, 813.
Magee, P. N. and Barnes, J. M.—(1956) *Brit. J. Cancer*, **10**, 114.
Nesbitt, Brenda, F., O'Kelly, J., Sargeant, K. and Sheridan, Ann.—(1962) *Nature, Lond.*, **195**, 1067.
Orr, J. W.—(1940) *J. Path. Bact.*, **50**, 393.
Paget, G. E.—(1954) *Ibid.*, **67**, 393.
Rogers, J. B. and Blumenthal, H. T.—(1960) *Cancer Res.*, **20**, 191.
Salmon, W. D. and Newberne, M.—(1963) *Ibid.*, **23**, 511.
Sargeant, K., Sheridan, Ann, O'Kelly, J. and Carnaghan, R. B. A.—(1961) *Nature, Lond.*, **192**, 1096.
Schoental, R.—(1961) *Brit. J. Cancer*, **15**, 812.
Sutton, P. M.—(1962) *Ibid.*, **16**, 619.
Uruguchi, J., Sakai, F., Tsukioka, M., Noguchi, Y., Tatsuno, T., Saito, M., Enomoto, M., Ishiko, T., Shikata, T. and Miyake, M.—(1961) *Jap. J. exp. Med.*, **31**, 435.